# Integrative multi-omics analysis identifies a robust 14-metabolite signature and reveals microbiome–metabolite–host interactions in atherosclerosis

**DOI:** 10.3389/fcvm.2026.1849138

**Published:** 2026-06-08

**Authors:** Leiyang Dai, Xiao Wang, Hui Zhang, Rong Gong, Jianli Pang, Junxi Pan, Qiuyue Xu, Yong Duan

**Affiliations:** Yunnan Key Laboratory of Laboratory Medicine, Yunnan Clinical Research Center for Laboratory Medicine, Department of Clinical Laboratory, the First Affiliated Hospital of Kunming Medical University, Kunming, Yunnan, China

**Keywords:** atherosclerosis, cellular senescence, gut microbiota, machine learning, metabolomics

## Abstract

**Background:**

Atherosclerosis (AS) is a complex metabolic and inflammatory disease in which interactions between host metabolism and gut microbiota play critical roles. However, robust metabolic biomarkers and their integration with microbial and host factors remain incompletely understood.

**Methods:**

We performed untargeted metabolomics to characterize metabolic alterations between AS patients and healthy controls (HC). Differential metabolites were identified and subjected to pathway enrichment analysis. Three machine learning models, including random forest (RF), least absolute shrinkage and selection operator (LASSO), and support vector machine (SVM), were applied to identify key metabolite signatures. Gut microbiota composition was analyzed using 16S rRNA sequencing, and correlation analyses were conducted to explore microbiome–metabolite interactions. In addition, inflammatory and senescence-related markers were assessed to evaluate host responses.

**Results:**

A total of 122 differential metabolites were identified between AS and HC, primarily enriched in amino acid–related pathways, including tryptophan, phenylalanine, and methionine metabolism. Machine learning integration revealed a robust panel of 14 overlapping metabolites with strong discriminative performance. Among them, Trimethylamine N-oxide, 3-Hydroxyhippuric acid, and Cholesteryl sulfate showed the highest diagnostic potential. Despite limited differences in gut microbial composition, several microbiota-derived metabolites and significant correlations between specific genera and metabolites were observed, suggesting functional alterations in the microbiome. Furthermore, senescence markers P16 and P21 were significantly elevated in AS and were associated with key metabolites and microbial taxa, whereas classical inflammatory markers showed no significant differences.

**Conclusion:**

This study identifies a robust metabolite signature associated with AS and highlights a coordinated microbiome–metabolite–host interaction network. These findings provide new insights into the metabolic mechanisms underlying AS and suggest potential biomarkers and therapeutic targets for disease diagnosis and intervention.

## Introduction

Atherosclerosis (AS) is a chronic, progressive vascular disease and the primary pathological basis of most cardiovascular events, including coronary artery disease and stroke (1, 2). Despite substantial advances in lipid-lowering therapies, the global burden of AS remains high, suggesting that mechanisms beyond traditional risk factors contribute to its development and progression (3, 4). Increasing evidence indicates that AS is not merely a lipid storage disease, but a complex systemic disorder involving metabolic dysregulation, immune activation, and vascular dysfunction (5–7).

Metabolic reprogramming has emerged as a critical feature of cardiovascular diseases (8). In particular, alterations in amino acid metabolism, lipid metabolism, and gut microbiota–derived metabolites have been implicated in endothelial dysfunction, oxidative stress, and chronic inflammation (9, 10). For example, metabolites such as Trimethylamine N-oxide (TMAO) and indoxyl sulfate, which are derived from gut microbial metabolism, have been shown to promote atherogenesis through multiple mechanisms (9, 11, 12). However, a comprehensive characterization of metabolic alterations in AS, especially at the systems level, remains incomplete.

High-throughput metabolomics provides a powerful tool to systematically profile metabolic changes and identify potential biomarkers. However, the high dimensionality and complexity of metabolomic data pose challenges for conventional statistical approaches (13). In recent years, machine learning methods, including random forest (RF), least absolute shrinkage and selection operator (LASSO), and support vector machine (SVM), have been increasingly applied to improve biomarker selection and disease classification (14–16). Integrating multiple machine learning algorithms may enhance the robustness and reproducibility of identified metabolic signatures.

In addition to host metabolism, the gut microbiota has been recognized as an important contributor to AS (17). While many studies have focused on compositional differences in microbial communities, emerging evidence suggests that functional outputs of the microbiome, particularly metabolite production, may play a more direct role in disease pathogenesis (18, 19). Therefore, integrating microbiome data with metabolomic profiles may provide deeper insights into host–microbe interactions in AS.

Furthermore, cellular senescence has recently been identified as a key driver of vascular aging and atherosclerotic progression (20, 21). Senescent cells accumulate in atherosclerotic plaques and contribute to inflammation and tissue remodeling (20, 22). However, the relationship between metabolic alterations, gut microbiota, and cellular senescence in AS remains poorly understood.

In this study, we performed an integrated analysis combining untargeted metabolomics, machine learning approaches, gut microbiota profiling, and experimental validation. We aimed to systematically characterize metabolic alterations associated with AS and to identify potential metabolite signatures with diagnostic value. In addition, we explored the relationships among gut microbiota, host metabolism, and cellular senescence to provide insights into the underlying mechanisms of AS.

Our findings provide a comprehensive view of metabolic dysregulation in AS and suggest a potential microbiome–metabolite–senescence axis, offering new insights into disease mechanisms and potential diagnostic biomarkers.

## Method

### Untargeted metabolomics analysis

Untargeted metabolomics analysis was performed on plasma samples using an ultra-high-performance liquid chromatography–mass spectrometry (UHPLC–MS/MS)-based platform. Plasma samples were thawed on ice and extracted using methanol/acetonitrile (1:1, v/v) containing isotopically labeled internal standards. Equal aliquots from all samples were pooled to generate quality control (QC) samples for monitoring analytical stability throughout the experiment. Chromatographic separation was performed using a Vanquish UHPLC system (Thermo Fisher Scientific, USA) equipped with a Waters ACQUITY UPLC BEH Amide column (2.1 mm × 50 mm, 1.7 μm). Mass spectrometry data were acquired using an Orbitrap Exploris 120 mass spectrometer operated in both positive and negative electrospray ionization modes. Raw LC–MS data were converted to mzXML format using ProteoWizard (v3.0.24054) and processed using in-house R scripts with the BiotreeDB database (v3.0) for metabolite annotation. Data preprocessing included noise filtering, missing value imputation, and total ion current (TIC)-based normalization. QC samples showed strong clustering and high inter-sample correlation, indicating good analytical reproducibility and data stability. Metabolite annotation confidence was classified according to the Metabolomics Standards Initiative (MSI) criteria. Only annotated metabolites with MSI levels 1–3 were included in downstream analyses.

### Differential metabolite analysis and visualization

Differential metabolite analysis was performed based on relative metabolite abundance data. Fold change (FC) between the two groups was calculated for each metabolite and transformed to log_2_ scale. Statistical significance was assessed using corresponding *P* values, and variable importance in projection (VIP) scores were obtained.

Metabolites were classified as significantly upregulated or downregulated based on the following criteria: VIP > 1, |log_2_FC| > 0.5, and *P* < 0.05. Due to the exploratory nature and relatively limited sample size of this study, unadjusted *P* values were initially used for metabolite screening in combination with VIP and fold change criteria. Metabolites not meeting these thresholds were considered non-significant.

A volcano plot was generated using the ggplot2 package in R (version 4.3.2), with log_2_(FC) on the *x*-axis and −log_10_(*P* value) on the *y*-axis. Different colors were used to distinguish upregulated, downregulated, and non-significant metabolites. The numbers of significantly upregulated and downregulated metabolites were annotated on the plot to provide an overview of the differential distribution.

### KEGG pathway enrichment analysis and visualization

Kyoto Encyclopedia of Genes and Genomes (KEGG) pathway enrichment analysis was conducted for the identified differential metabolites. The number of metabolites enriched in each pathway (hits) and their corresponding statistical significance were calculated.

The enrichment results were visualized using both bar plots and bubble plots. In the bar plot, pathways were ranked based on the number of enriched metabolites, and a color gradient was applied to represent the enrichment level. The number of hits was additionally labeled on each bar.

For the bubble plot, pathways were displayed on the *y*-axis and background metabolite counts on the *x*-axis. Bubble size represented the number of hits, while color indicated the significance level (*P* value), allowing for an integrated visualization of enrichment magnitude and statistical relevance.

### Machine learning–based feature selection and identification of key metabolites

To identify candidate metabolic biomarkers, three machine learning algorithms, including Random Forest (RF), Support Vector Machine (SVM), and Least Absolute Shrinkage and Selection Operator (LASSO), were applied to the differential metabolite dataset.

For all models, metabolite abundance data were log-transformed and standardized prior to analysis. Samples were labeled as a binary outcome (AS vs. HC). A five-fold cross-validation strategy was employedfor internal model validation to evaluate model performance and reduce overfitting. Model performance was assessed by receiver operating characteristic (ROC) curves and the area under the curve (AUC). In addition, classification performance was evaluated using confusion matrix–derived metrics, including accuracy, sensitivity, specificity, and balanced accuracy.

The RF model was constructed using the *randomForest* package with 500 trees, and feature importance was derived based on mean decrease in accuracy. The SVM model was implemented using a linear kernel with a regularization parameter (cost) of 0.1. The LASSO model was built using the *glmnet* package with binomial regression, and the optimal penalty parameter (*λ*) was determined via internal cross-validation.

SHAP analysis was used as an interpretative approach for feature importance ranking rather than as an independent validation method. For each model, the top 15 metabolites were selected based on SHAP importance ranking.

To obtain a set of candidate metabolites consistently identified across multiple algorithms, the top-ranked metabolites from the three models were integrated. An UpSet analysis was performed to assess the overlap among RF, SVM, and LASSO results. Metabolites appearing in at least two of the three models were retained as candidate metabolites. This approach prioritized metabolites reproducibly identified across multiple machine learning methods.

Finally, a total of 15 metabolites that met this criterion were defined as key metabolites. These metabolites were further evaluated using single-metabolite ROC analysis to assess their individual discriminative ability.

### 16S rRNA gene sequencing and microbiome analysis

#### Alpha and beta diversity analysis

Alpha diversity was assessed to evaluate within-sample microbial richness and diversity. Common indices, including Shannon, observed_features, Simpson, and Chao1, were calculated using the R package *vegan*. Differences in alpha diversity between groups were compared using the Wilcoxon rank-sum test.

Beta diversity was used to assess differences in microbial community structure between samples. Bray–Curtis distance matrices were calculated, and principal coordinates analysis (PCoA) was performed for visualization. Statistical significance of group separation was evaluated using permutational multivariate analysis of variance (PERMANOVA).

#### Taxonomic composition analysis

The relative abundances of microbial taxa at the phylum and genus levels were calculated and visualized. Stacked bar plots were generated to display the composition and distribution of dominant taxa across samples and groups. Taxa with the highest relative abundance were selected to represent the microbial community structure.

#### Differential abundance analysis

To identify taxa with differential abundance between groups, the Wilcoxon rank-sum test was applied at the genus level. *P*-values were adjusted for multiple testing using the Benjamini–Hochberg method. Taxa with an adjusted *P* value <0.05 were considered statistically significant.

### Functional prediction analysis (PICRUSt2)

Microbial functional profiles were inferred from 16S rRNA gene sequencing data using *PICRUSt2* (Phylogenetic Investigation of Communities by Reconstruction of Unobserved States). The predicted gene family abundances were annotated against the Kyoto Encyclopedia of Genes and Genomes (KEGG) database. Subsequently, functional pathways were categorized at KEGG level 2, and their relative abundances were calculated for each sample. Differences in functional profiles between groups were assessed and visualized using bar plots to compare the distribution of major metabolic pathways.

### Correlation analysis between microbiota, metabolites, and senescence markers

Spearman correlation analysis was performed to evaluate the associations among key metabolites, gut microbiota, and senescence-related markers. A total of 14 key metabolites identified from machine learning analyses and the top 15 genera based on relative abundance were included. Correlation coefficients were calculated using the Spearman rank method to account for non-normal data distribution.

For each pairwise comparison, corresponding *P*-values were computed, and statistical significance was defined as *P* < 0.05. Correlation matrices were visualized as heatmaps, in which the color scale represented the strength and direction of the correlation (yellow for positive correlations and cyan for negative correlations). Significant correlations were annotated with asterisks (*P* < 0.05, *P* < 0.01).

In addition, correlations between senescence-related markers (P16 and P21) and both metabolites and microbial genera were further assessed using the same approach. All analyses were performed in R, primarily using the Hmisc package (v_5.2-5) for correlation calculation and corrplot package (v_0.95) for visualization.

### Quantitative PCR (qPCR) analysis

Total RNA was extracted from peripheral blood mononuclear cells (PBMCs) using the TRIzol reagent according to the manufacturer's instructions. The RNA concentration and purity were assessed using a NanoDrop 2000 spectrophotometer. Complementary DNA (cDNA) was synthesized from 1 μg of total RNA using the PrimeScript RT Reagent Kit. Quantitative PCR was performed on a CFX96 real-time PCR system using SYBR Green Master Mix. The cycling conditions were: 95 °C for 30 s, followed by 40 cycles of 95 °C for 5 s and 60 °C for 30 s. Relative gene expression levels were calculated using the 2^–ΔΔCt^ method and normalized to GAPDH as an internal control. Target genes included P16, P21, P65, and Caspase-1. All samples were run in triplicate to ensure reproducibility. The primer information is recorded in Table 1.

**Table 1:** qPCR primer information.

Primer	Sequence (5’ to 3’)
P16-F	CACTCTCACCCGACCCGT
P16-R	GGTCCACGGGCAGACG
P21-F	CTCAAATCGTCCAGCGACCT
P21-R	GACTCCTTGTTCCGCTGCTA
Caspase-1-F	CCAGATGGTAGAGCGCAGAT
Caspase-1-R	CTGCCCACAGACATTCATACAG
p65-F	GTCCCTGTGCCTAACACCAG
p65-R	GTCCCAGACCAAACCCCTTC
GAPDH-F	GGACCTGACCTGCCGTCTAG
GAPDH-R	GTAGCCCAGGATGCCCTTGA

### Enzyme-linked immunosorbent assay (ELISA)

Plasma levels of IL-6, IL-18, and MCP-1 were measured using commercially available ELISA kits according to the manufacturer's instructions. Briefly, 100 μL of standards or samples were added to 96-well plates pre-coated with capture antibodies and incubated at room temperature for 2 h. After washing, 100 μL of biotinylated detection antibody was added and incubated for 1 h, followed by the addition of 100 μL of streptavidin–horseradish peroxidase (HRP) for 30 min. The reaction was developed using tetramethylbenzidine (TMB) substrate and stopped with 2N sulfuric acid. Absorbance was measured at 450 nm using a microplate reader. All measurements were performed in duplicate, and concentrations were calculated from standard curves.

### Statistical analysis

All statistical analyses were performed using R software (version 4.3.2). Continuous variables were expressed as mean ± standard deviation or median (interquartile range) as appropriate. Group comparisons were conducted using Student's *t*-test or Wilcoxon rank-sum test depending on data distribution. Categorical variables were compared using Fisher's exact test when applicable.

For metabolomics data, an integrated analytical workflow was applied. Initial feature screening was performed using fold change (FC), variable importance in projection (VIP), and unadjusted *P* values due to the exploratory nature of this study and limited sample size. This univariate screening was followed by multivariate and machine learning–based feature selection to ensure robustness of metabolite identification.

To evaluate classification performance and reduce overfitting risk, a five-fold cross-validation strategy was used in all machine learning models (Random Forest, Support Vector Machine, and LASSO regression). Receiver operating characteristic (ROC) curves and area under the curve (AUC) were used to assess model performance. Additional evaluation metrics included accuracy, sensitivity, specificity, and balanced accuracy derived from confusion matrices.

Feature importance was further interpreted using Shapley Additive Explanations (SHAP), which served as an explanatory tool rather than an independent validation method. Metabolites consistently identified across at least two machine learning algorithms were defined as candidate biomarker candidates for downstream analysis.

For microbiome and functional prediction analyses, alpha diversity was assessed using Shannon, Simpson, Chao1, and observed features indices, and compared using Wilcoxon rank-sum tests. Beta diversity differences were evaluated using Bray–Curtis distance and PERMANOVA analysis.

Correlation analyses among metabolites, microbial taxa, and clinical markers were performed using Spearman correlation coefficients. All *P* values were two-sided, and statistical significance was defined as *P* < 0.05. No adjustment for multiple testing was applied in exploratory correlation analyses.

This study adopts an exploratory, multi-step analytical framework integrating univariate statistics, multivariate modeling, and machine learning–based feature selection. Findings should therefore be interpreted as hypothesis-generating rather than confirmatory and require validation in independent external cohorts.

## Results

### Baseline clinical and biochemical characteristics of the study cohort

Baseline clinical and biochemical characteristics of participants are summarized in Table 2. A total of 20 subjects were included, comprising 10 atherosclerosis (AS) patients and 10 healthy controls (HC). Compared with the HC group, AS patients were significantly older (60.5 [55.8–71.8] vs. 50.0 [44.3–52.0] years, *P* = 0.002) and exhibited a higher BMI (24.43 [23.29–25.91] vs. 22.80 [21.13–23.51], *P* = 0.043). In addition, hypertension was more prevalent in the AS group (70% vs. 10%, *P* = 0.022). Among lipid-related parameters, total cholesterol (TC) was lower in AS patients compared with controls (2.47 [1.20–3.33] vs. 6.03 [5.25–6.13], *P* = 0.161), while no significant differences were observed in triglycerides (TG), low-density lipoprotein (LDL), or high-density lipoprotein (HDL). Uric acid (UA) levels were significantly higher in the AS group than in controls (384.05 [337.90–432.47] vs. 307.55 [284.90–334.40], *P* = 0.029). No statistically significant differences were observed in fasting blood glucose (FBG), blood urea nitrogen (BUN), creatinine (CR), aspartate aminotransferase (AST), alanine aminotransferase (ALT), or C-reactive protein (CRP) between the two groups.

**Table 2:** Baseline clinical and biochemical characteristics of study participants.

Variable	Level	HC	AS	*P*	Test	Signif
Age		50 (44.25–52)	60.5 (55.75–71.75)	0.002176912	Wilcoxon rank-sum test	**
Sex	Female	5 (50.0)	4 (40.0)	1	Fisher's exact test	
	Male	5 (50.0)	6 (60.0)			
BMI		22.8 (21.13–23.51)	24.43 (23.29–25.91)	0.043257053	Wilcoxon rank-sum test	*
Alcohol		2 (20%)	1 (10%)	1	Fisher's exact test	
Smoking	No	8 (80.0)	5 (50.0)	0.348	Fisher's exact test	
	Yes	2 (20.0)	5 (50.0)			
Hypertension	No	9 (90.0)	3 (30.0)	0.022	Fisher's exact test	*
	Yes	1 (10.0)	7 (70.0)			
FBG		4.44 (4.28–4.74)	5.38 (4.62–7.2)	0.11524172	Wilcoxon rank-sum test	
Diabetes	No	10 (100.0)	9 (90.0)	1	Fisher's exact test	
	Yes	0 (0.0)	1 (10.0)			
TC		6.03 (5.25–6.13)	2.47 (1.2–3.33)	0.160839161	Wilcoxon rank-sum test	
TG		3.14 (2.38–3.79)	3.3 (2.92–3.96)	0.937062937	Wilcoxon rank-sum test	
LDL		2.78 (2.56–3.42)	1.73 (1.44–2.72)	0.160839161	Wilcoxon rank-sum test	
HDL		1.03 (0.94–1.08)	0.9 (0.84–1.02)	0.573426573	Wilcoxon rank-sum test	
UA		307.55 (284.9–334.4)	384.05 (337.9–432.47)	0.02880556	Wilcoxon rank-sum test	*
BUN		5.76 (4.76–6.68)	6.42 (5.46–7.04)	0.242806729	Wilcoxon rank-sum test	
CR		66.55 (48.32–73.73)	76.6 (67.8–92.8)	0.094719522	Wilcoxon rank-sum test	
AST		27.5 (24.35–33.4)	24.6 (20.4–27.8)	0.242806729	Wilcoxon rank-sum test	
ALT		23.3 (14.08–31.35)	23.7 (19.2–26.6)	0.743863052	Wilcoxon rank-sum test	
CRP		4.4 (2.48–8.73)	0.92 (0.7–1.97)	0.171428571	Wilcoxon rank-sum test	

*P < 0.05, **P < 0.01.

Overall, the two groups were generally comparable in most metabolic and inflammatory parameters, with differences mainly observed in age, BMI, hypertension status, and uric acid levels.

### Distinct metabolic profiles and identification of differential metabolites between AS and HC groups

**Figure 1:**
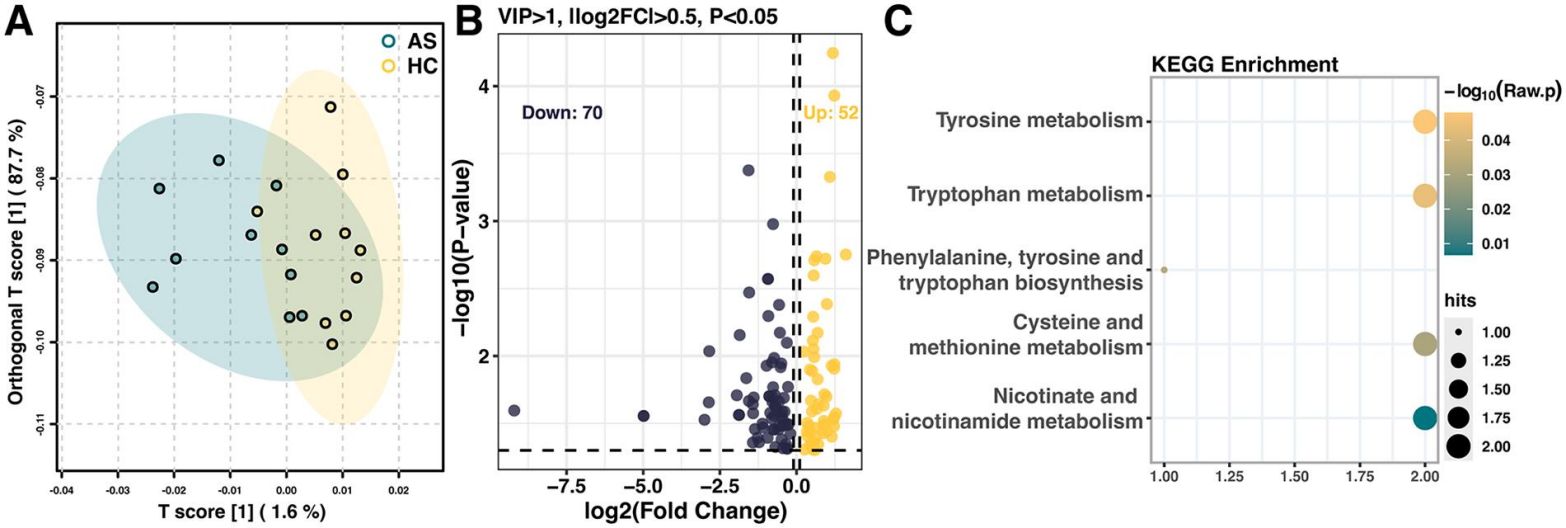
Metabolic profiling and pathway enrichment analysis between AS and HC groups. **(A)** OPLS-DA score plot showing clear separation between AS and HC groups, indicating distinct metabolic patterns (*T* score [1] = 1.6%, Orthogonal *T* score [1] = 87.7%). **(B)** Volcano plot of differential metabolites identified based on VIP > 1, |log_2_FC| > 0.5, and *P* < 0.05, with 52 upregulated and 70 downregulated metabolites. **(C)** KEGG pathway enrichment analysis of differential metabolites, highlighting significantly enriched pathways, including tyrosine metabolism, tryptophan metabolism, phenylalanine, tyrosine and tryptophan biosynthesis, cysteine and methionine metabolism, and nicotinate and nicotinamide metabolism (*P* < 0.05).

To investigate metabolic alterations between the AS and HC groups, an orthogonal partial least squares discriminant analysis (OPLS-DA) model was constructed. The two groups were clearly separated, indicating distinct metabolic profiles. The model showed a *T* score [1] of 1.6% and an orthogonal *T* score [1] of 87.7%, suggesting that the variation between groups was well captured by the model (Figure 1A). Differential metabolite analysis was performed using the criteria of VIP > 1, |log_2_ fold change| > 0.5, and *P* < 0.05. A total of 122 metabolites were identified as significantly altered, including 52 upregulated and 70 downregulated metabolites in the AS group compared with the HC group (Figure 1B). To further explore the biological significance of these metabolites, KEGG pathway enrichment analysis was conducted. The results demonstrated that differential metabolites were significantly enriched in several amino acid-related metabolic pathways, including tyrosine metabolism, tryptophan metabolism, phenylalanine, tyrosine and tryptophan biosynthesis, cysteine and methionine metabolism, as well as nicotinate and nicotinamide metabolism (all *P* < 0.05; Figure 1C).

### Random forest (RF) model analysis identified key metabolite signatures distinguishing AS from HC

**Figure 2:**
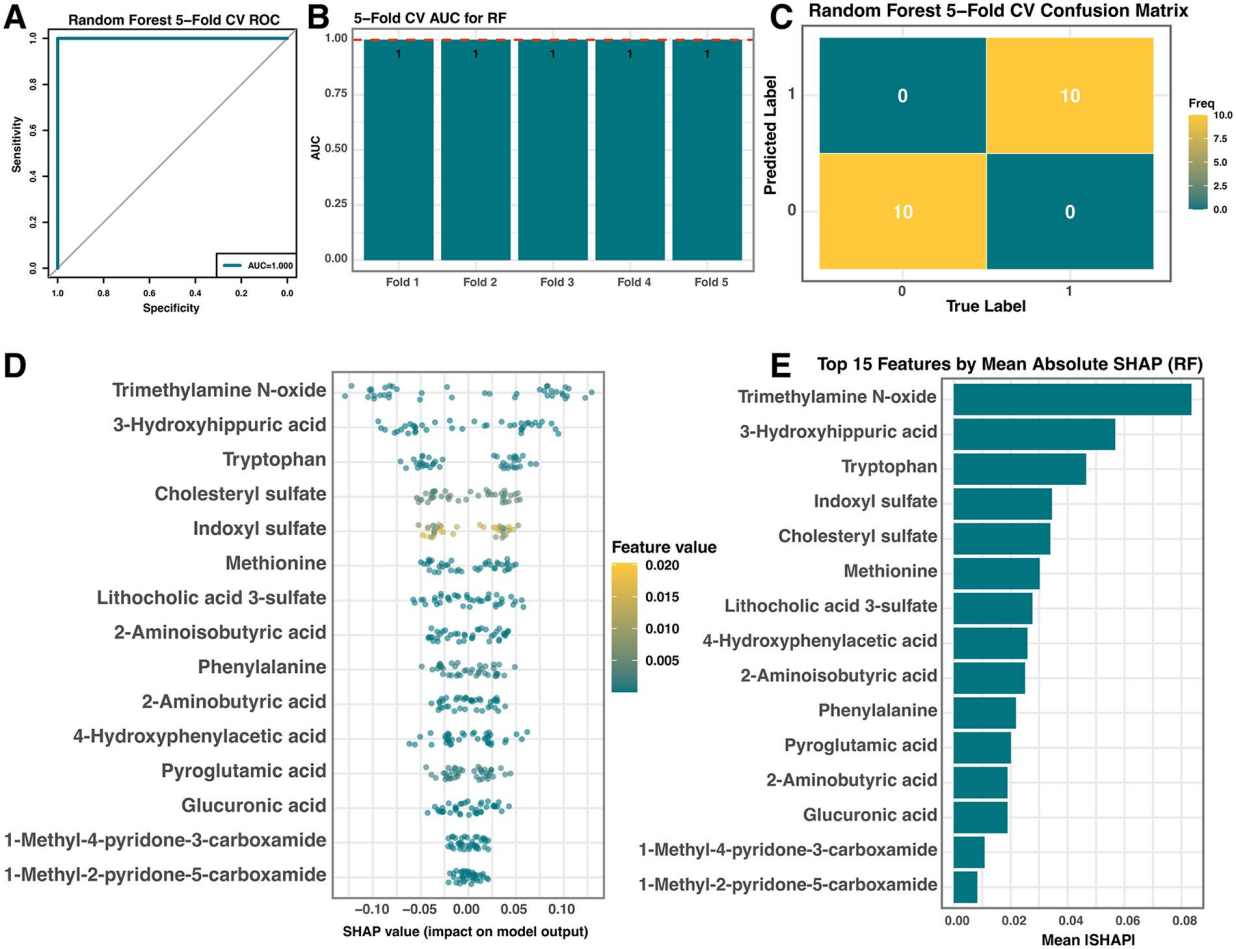
Random forest model identification and interpretation of key differential metabolites. **(A)** Receiver operating characteristic (ROC) curve of the random forest model based on 5-fold cross-validation. **(B)** AUC values for each fold in the 5-fold cross-validation, demonstrating consistent model performance. **(C)** Confusion matrix of the random forest model, indicating classification performance across AS and HC samples. **(D)** SHAP summary (beeswarm) plot showing the contribution of each metabolite to the model. Features are ranked by importance, and colors represent feature values (yellow: high; cyan: low). **(E)** Bar plot of the top 15 metabolites ranked by mean absolute SHAP values, highlighting the most influential metabolic features driving classification.

To further evaluate the diagnostic potential of differential metabolites, a random forest (RF) model was constructed based on all 122 differential metabolites identified between AS and HC. The RF model achieved excellent discrimination between the two groups, with a 5-fold cross-validated ROC curve yielding an AUC of 1.0. Consistently, all five folds reached an AUC value of 1.0, indicating highly stable model performance in the internal cross-validation setting (Figures 2A,B). This performance was obtained based on internal cross-validation without independent external validation.

The confusion matrix further demonstrated the robustness of the model, with perfect classification of all samples (accuracy = 1.0, sensitivity = 1.0, specificity = 1.0; Figure 2C). These results suggest that the selected metabolite features have strong predictive power.

To interpret the contribution of individual metabolites, SHAP (Shapley Additive Explanations) analysis was performed. The SHAP summary plot ranked metabolites according to their impact on model output. Among them, Trimethylamine N-oxide, 3-Hydroxyhippuric acid, Tryptophan, and Cholesteryl sulfate were identified as the most influential features. Notably, Indoxyl sulfate showed a distinct distribution pattern, with higher feature values predominantly contributing to classification, whereas most other metabolites exhibited lower feature values driving the model (Figure 2D).

The SHAP-based feature importance ranking further confirmed these findings. The top contributors, ranked by mean absolute SHAP values, included Trimethylamine N-oxide, 3-Hydroxyhippuric acid, Tryptophan, Indoxyl sulfate, and Cholesteryl sulfate, followed by Methionine and Lithocholic acid 3-sulfate (Figure 2E). These metabolites may represent key metabolic signatures associated with AS.

### LASSO model identifies robust metabolite predictors with high classification performance

**Figure 3:**
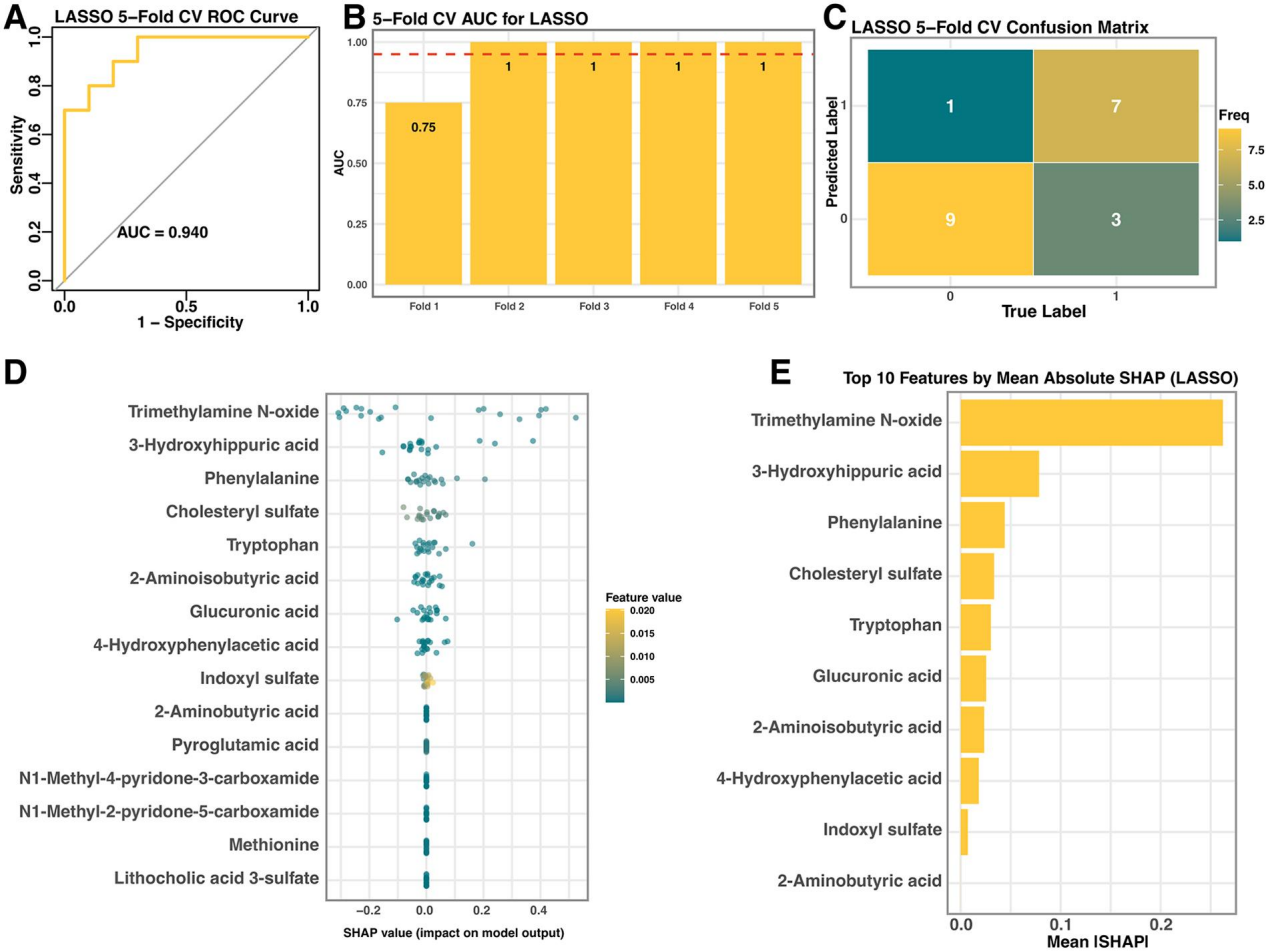
LASSO model performance and identification of key metabolites distinguishing AS from HC. **(A)** Receiver operating characteristic (ROC) curve of the LASSO model based on 5-fold cross-validation. **(B)** AUC values for each fold in the 5-fold cross-validation, demonstrating generally stable model performance. **(C)** Confusion matrix of the LASSO model. **(D)** SHAP beeswarm plot showing the contribution of individual metabolites to the model prediction. Features are ranked from top to bottom according to their importance, and colors represent feature values (yellow: high; blue: low). **(E)** Bar plot of the top metabolites ranked by mean absolute SHAP values, indicating their relative importance in the LASSO model.

To further validate the predictive value of differential metabolites, a least absolute shrinkage and selection operator (LASSO) model was constructed based on the same set of 122 differential metabolites. The LASSO model demonstrated strong discrimination between AS and HC, with a 5-fold cross-validated ROC curve yielding an overall AUC of 0.94 in internal validation (Figure 3A).

The performance across individual folds was generally stable, with four folds achieving an AUC of 1.0 and one fold showing a slightly lower AUC of 0.75 (Figure 3B). Consistent with this, the confusion matrix indicated an overall classification accuracy of 0.80, with a sensitivity of 0.90 and a specificity of 0.70 (Figure 3C), suggesting a relatively high ability to correctly identify AS samples while maintaining moderate specificity.

SHAP analysis was further applied to interpret the contribution of individual metabolites to the LASSO model. The SHAP beeswarm plot revealed that key metabolites contributing to model prediction included Trimethylamine N-oxide, 3-Hydroxyhippuric acid, Phenylalanine, Cholesteryl sulfate, and Tryptophan, among others (Figure 3D). These metabolites exhibited distinct SHAP value distributions, indicating their differential influence on classification outcomes.

In addition, ranking of features based on mean absolute SHAP values identified the top contributors to the model (Figure 3E). The most influential metabolites included Trimethylamine N-oxide, 3-Hydroxyhippuric acid, and Phenylalanine, followed by Cholesteryl sulfate, Tryptophan, and Glucuronic acid, highlighting their potential importance in distinguishing AS from HC.

### Support vector machine model further confirms the discriminative metabolic signature between AS and HC

**Figure 4:**
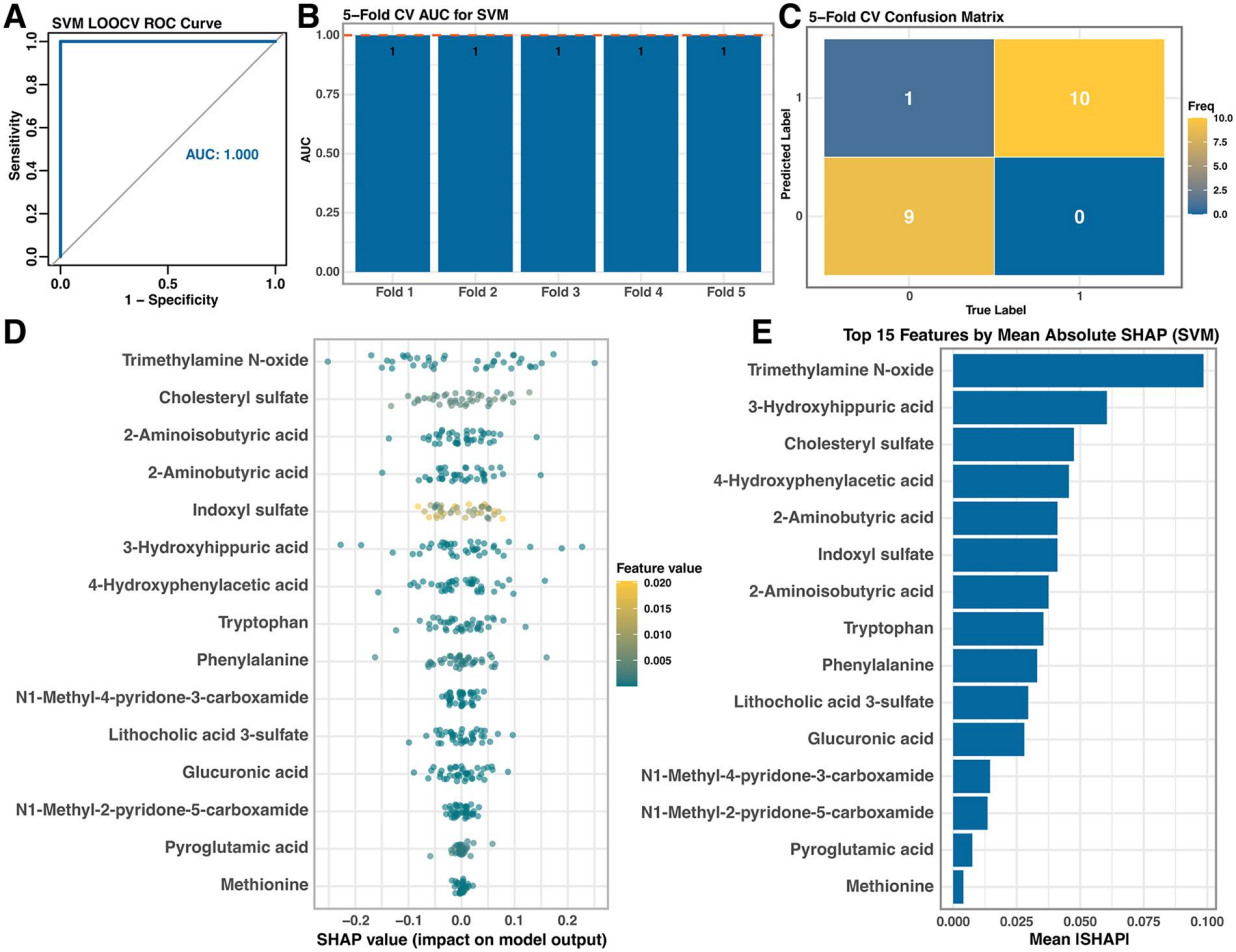
SVM model performance and identification of key metabolic features distinguishing AS from HC. **(A)** Receiver operating characteristic (ROC) curve of the SVM model based on 5-fold cross-validation. **(B)** AUC values for each fold in the 5-fold cross-validation, demonstrating consistently perfect model performance. **(C)** Confusion matrix of the SVM model, showing classification results across AS and HC samples. **(D)** SHAP beeswarm plot illustrating the contribution of each metabolite to the SVM model. Features are ranked from top to bottom based on importance, and colors represent feature values. **(E)** Bar plot of the top 15 metabolites ranked by mean absolute SHAP values, indicating their relative importance in the SVM model.

To further validate the robustness of the identified metabolic signatures, a support vector machine (SVM) model was constructed based on the same set of 122 differential metabolites. The SVM model achieved excellent classification performance, with the receiver operating characteristic (ROC) curve yielding an AUC of 1.0 under leave-one-out cross-validation (LOOCV) within the current dataset (Figure 4A).

Consistently, the 5-fold cross-validation analysis showed perfect performance across all folds, with each fold achieving an AUC of 1.0 (Figure 4B), indicating high stability of the model. However, the confusion matrix revealed imbalanced classification results (Figure 4C), suggesting potential bias in prediction despite the high AUC.

To further interpret the model, SHAP analysis was performed. The SHAP beeswarm plot ranked metabolites according to their contribution to model prediction (Figure 4D). Among them, Trimethylamine N-oxide, Cholesteryl sulfate, and 2-Aminoisobutyric acid were identified as the most influential features. Additional metabolites, including Indoxyl sulfate, 3-Hydroxyhippuric acid, and Tryptophan, also contributed to model performance.

Feature importance analysis based on mean absolute SHAP values further confirmed these findings (Figure 4E). The top-ranked metabolites included Trimethylamine N-oxide, 3-Hydroxyhippuric acid, and Cholesteryl sulfate, followed by 4-Hydroxyphenylacetic acid, 2-Aminobutyric acid, and Indoxyl sulfate, indicating that these metabolites play key roles in distinguishing AS from HC.

### Integrated machine learning analysis identified robust metabolite signatures with diagnostic potential

**Figure 5:**
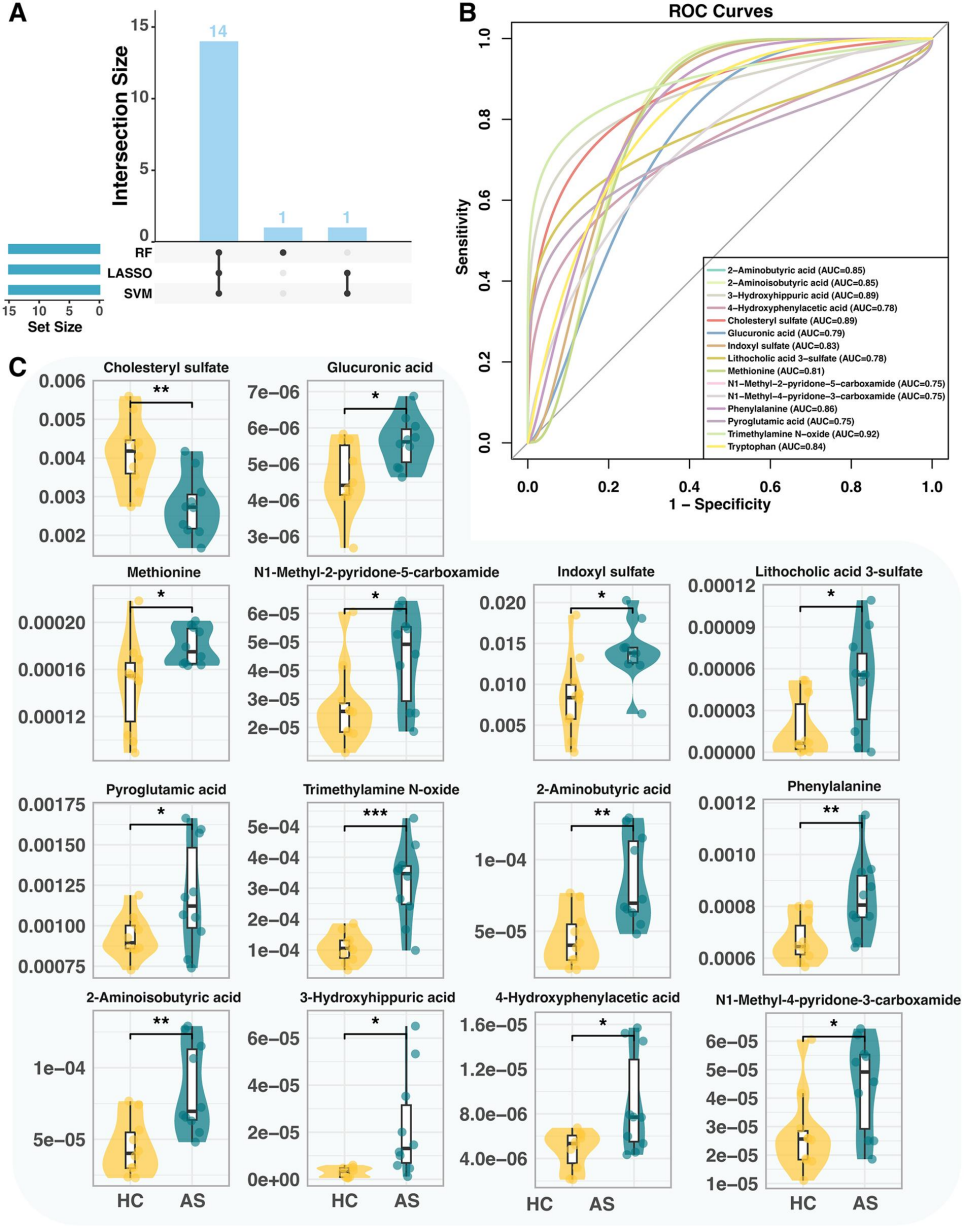
Integrated identification and validation of key metabolic biomarkers. **(A)** UpSet plot showing the overlap of the top 15 metabolites identified by RF, LASSO, and SVM models. Fourteen metabolites were shared across all three models. **(B)** ROC curve analysis of the overlapping metabolites, demonstrating their diagnostic performance in distinguishing AS from HC. **(C)** Comparison of key metabolite levels between AS and HC groups. Violin plots display the distribution of the data, boxplots indicate the median and interquartile range (IQR), and overlaid dots represent individual samples. Whiskers indicate the data range within 1.5 × IQR. Statistical significance was assessed using Student's *t*-test (*P* < 0.05:*, *P* < 0.01:**).

To identify candidate metabolic biomarkers, the top 15 metabolites selected by the random forest (RF), LASSO, and SVM models were integrated. 14 metabolites were consistently identified across all three models, while only one metabolite was uniquely selected by the RF model and one metabolite overlapped between the LASSO and SVM models (Figure 5A). This high degree of overlap suggests consistency of the identified metabolic signatures within the current cohort and internal validation framework.

To further evaluate their diagnostic potential, receiver operating characteristic (ROC) curve analysis was performed within the same cohort for the 14 overlapping metabolites (Figure 5B). Most metabolites exhibited good discriminatory ability, with AUC values ranging from 0.75 to 0.92. Among them, Trimethylamine N-oxide showed the highest diagnostic performance (AUC = 0.92), followed by 3-Hydroxyhippuric acid and Cholesteryl sulfate (AUC = 0.89), as well as Phenylalanine (AUC = 0.86) and Tryptophan (AUC = 0.84).

Further comparison of metabolite levels between groups revealed that most metabolites were significantly elevated in the AS group compared with HC (Figure 5C). In contrast, Cholesteryl sulfate was significantly higher in the HC group, suggesting a potential protective or inversely associated role. Notably, Tryptophan did not show a statistically significant difference between groups despite its relatively high diagnostic performance. Which may require further validation in independent cohorts.

### Gut microbial composition and functional potential showed limited differences between AS and HC

**Figure 6:**
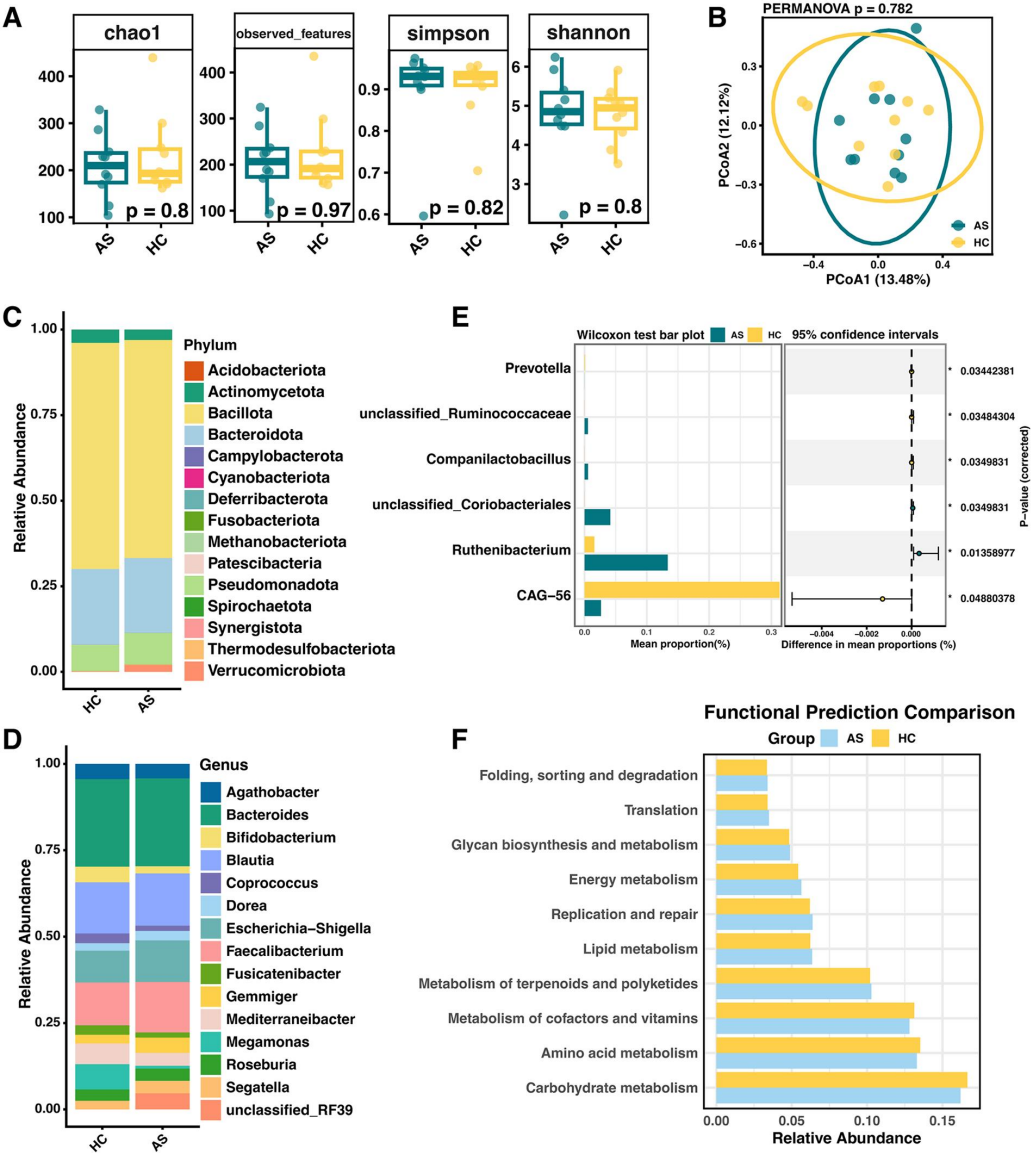
Gut microbiota diversity, composition, and functional prediction analysis. **(A)** Comparison of α-diversity indices (Chao1, observed features, Simpson, and Shannon) between AS and HC groups. Data are presented as median and interquartile range (IQR). Statistical significance was assessed using the Wilcoxon rank-sum test. No significant differences were observed. **(B)** β-diversity analysis based on principal coordinates analysis (PCoA), showing no clear separation between groups. Statistical significance was evaluated using PERMANOVA analysis. **(C)** Relative abundance of the top phylum in AS and HC groups. **(D)** Relative abundance of the top genus in AS and HC groups. **(E)** Differential genera identified using the Wilcoxon rank-sum test. **(F)** Functional prediction analysis based on KEGG level 2 pathways, showing differences in predicted metabolic functions between groups.

To explore gut microbiota alterations between AS and HC, both α-diversity and β-diversity were assessed. As shown in Figure 6A, no significant differences were observed in α-diversity indices between the two groups. Specifically, Chao1 richness was slightly higher in HC (*P* = 0.80), whereas observed features, Simpson index, and Shannon index were marginally higher in AS (*P* = 0.97, 0.82, and 0.80, respectively), indicating comparable microbial diversity.

Consistent with this, β-diversity analysis based on principal coordinates analysis (PCoA) did not reveal clear separation between AS and HC (Figure 6B). The first two principal coordinates explained 13.48% and 12.12% of the total variance, respectively, and PERMANOVA analysis confirmed no significant difference between groups (*P* = 0.782). These findings suggest that gut microbial alterations in AS are not primarily driven by large-scale compositional shifts.

At the taxonomic level, the overall microbial composition was broadly similar between groups. At the phylum level, Actinomycetota, Bacillota, Bacteroidota, and Pseudomonadota were the dominant phyla in both AS and HC (Figure 6C). At the genus level, several taxa showed relatively higher abundance in AS, including Escherichia–Shigella, Faecalibacterium, unclassified_RF39, and Gemmiger (Figure 6D). These subtle compositional differences indicate that specific microbial taxa rather than global community restructuring may contribute to metabolic alterations in AS.

Further differential analysis using the Wilcoxon test identified several genera with nominal significance, including Prevotella, unclassified_Ruminococcaceae, Companilactobacillus, unclassified_Coriobacteriales, Ruthenibacterium, and CAG-56 (*P* < 0.05). Among these, unclassified_Ruminococcaceae, Companilactobacillus, unclassified_Coriobacteriales, and Ruthenibacterium showed higher mean proportions in AS compared with HC (Figure 6E). These findings suggest that low-abundance but functionally relevant taxa may be involved in host metabolic modulation.

Functional prediction analysis revealed differences in microbial metabolic potential between groups (Figure 6F). Compared with HC, AS samples showed relatively higher abundance of pathways related to translation, glycan biosynthesis and metabolism, energy metabolism, replication and repair, lipid metabolism, and metabolism of terpenoids and polyketides, suggesting potential functional alterations despite limited compositional differences. Overall, these results indicate that gut microbiota in AS may undergo functional remodeling despite limited compositional differences. These functional alterations, particularly in pathways related to energy metabolism, lipid metabolism, and genetic information processing, may contribute to host metabolic homeostasis and disease progression.

### Validation of inflammatory markers and integrated microbiome–metabolite associations in AS

To further evaluate inflammatory status, qPCR and ELISA analyses were performed. As shown in Supplementary Figure S1A, the mRNA expression levels of P65 and Caspase-1 were higher in the AS group compared with HC, although these differences did not reach statistical significance (both *P* > 0.05), indicating limited alteration of classical inflammatory signaling in circulating PBMCs in this cohort. Similarly, ELISA results (Supplementary Figure S1B) showed that IL-18 and IL-6 tended to be elevated in the HC group, while MCP-1 exhibited a higher level in AS. However, none of these differences were statistically significant (all *P* > 0.05), suggesting that systemic inflammatory cytokines did not show robust group discrimination in the current dataset.

**Figure 7:**
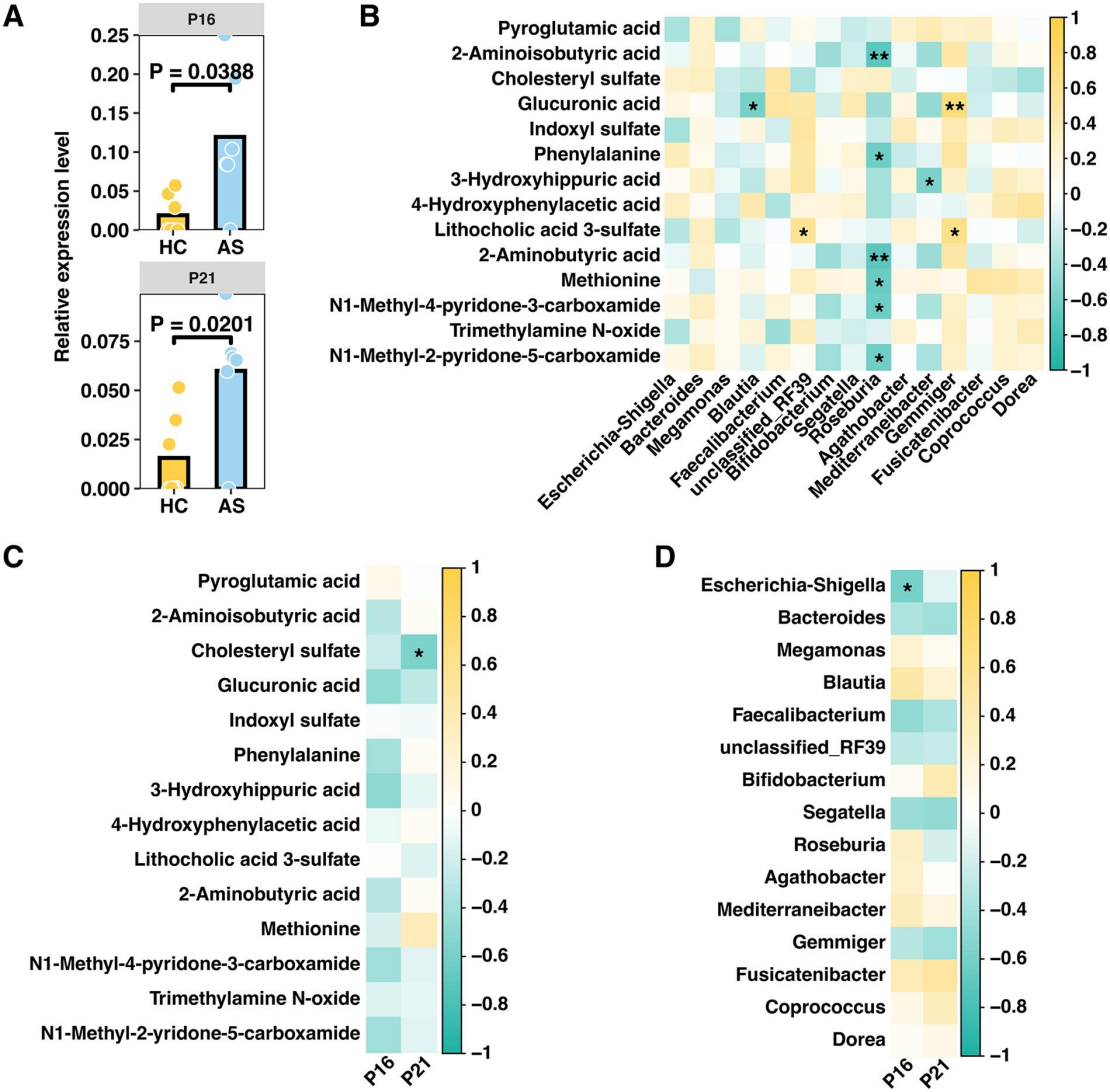
Integrated analysis of senescence markers, metabolites, and gut microbiota. **(A)** Relative mRNA expression levels of P16 and P21 in PBMCs, both significantly increased in AS compared with HC. Bars represent group mean, and overlaid dots represent individual samples. Statistical significance was assessed using Student's *t*-test, with exact *P* values indicated in the figure. **(B)** Spearman correlation heatmap between 14 key metabolites and the top 15 genera. Asterisks indicate statistical significance (*P < 0.05; **P < 0.01). **(C)** Correlation heatmap between P16/P21 and the 14 key metabolites. **(D)** Correlation heatmap between P16/P21 and the top 15 genera.

In contrast, senescence-related markers showedmore pronounced changes. As presented in Figure 7A, the expression levels of P16 and P21 were significantly increased in AS compared with HC, suggesting senescence-associated transcriptional alterations in circulating PBMCs, which may reflect systemic cellular stress rather than direct vascular cellular senescence (Supplementary Figure S1A), rather than direct evidence of cascular or tissue-level cellular senescence.

To explore the interplay between gut microbiota and host metabolism, Spearman correlation analysis was performed between the 14 key metabolites and the top 15 genera (Figure 7B). Several significant associations were identified. Glucuronic acid was negatively correlated with Blautia, whereas Lithocholic acid 3-sulfate showed a positive correlation with unclassified_RF39. Notably, Roseburia exhibited significant negative correlations with multiple metabolites, including N1-Methyl-2-pyridone-5-carboxamide, N1-Methyl-4-pyridone-3-carboxamide, 2-Aminobutyric acid, Methionine, Phenylalanine, and 2-Aminoisobutyric acid. In addition, Mediterraneibacter was negatively associated with 3-Hydroxyhippuric acid, while Gemmiger showed positive correlations with both Lithocholic acid 3-sulfate and Glucuronic acid. These findings reflect associative relationships rather than causal regulatory links.

Further correlation analyses revealed potential links between senescence markers and metabolic alterations. As shown in Figure 7C, P21 was significantly negatively correlated with Cholesteryl sulfate. Moreover, microbiome–host interactions were also observed at the bacterial level (Figure 7D), where P16 was negatively correlated with Escherichia–Shigella. Given the cross-sectional design and lack of functional validation, these associations should be interpreted as correlative signals requiring further confirmation.

Together, these results suggest coordinated alterations among gut microbiota, host metabolism, and senescence-associated transcriptional changes in circulating immune cells in AS.

## Discussion

### Metabolic reprogramming is a hallmark of AS

Atherosclerosis (AS) is increasingly recognized as a systemic metabolic disorder characterized by complex interactions among lipid metabolism, immune regulation, and vascular biology (5, 23, 24). In this study, we observed a clear separation of metabolic profiles between AS and healthy controls (HC), accompanied by the identification of 122 differential metabolites. Pathway enrichment analysis revealed that these metabolites were predominantly involved in amino acid–related metabolic pathways, including tryptophan, phenylalanine, tyrosine, and cysteine/methionine metabolism. Accumulating evidence suggests that disturbances in these pathways contribute to cardiovascular pathology (25–27). Tryptophan metabolism, particularly through the kynurenine pathway, has been linked to immune activation and endothelial dysfunction (28, 29), while methionine metabolism plays a critical role in redox balance with vascular aging (30). Similarly, elevated phenylalanine levels have been associated with increased cardiovascular risk and inflammatory responses (31, 32). These findings indicate that metabolic reprogramming in AS extends beyond lipid dysregulation and involves coordinated alterations in amino acid metabolism that may influence oxidative stress and immune homeostasis.

### Robust metabolite signatures identified by machine learning integration

To improve the reliability of biomarker identification, we applied three independent machine learning approaches (RF, LASSO, and SVM) and identified a consensus panel of 14 metabolites. The high degree of overlap across models highlights the consistency of these signatures within the current cohort and internal validation framework, reducing the likelihood of model-specific bias.

Although RF and SVM achieved near-perfect classification performance, in internal cross-validation, such results should be interpreted cautiously given the relatively small sample size and lack of independent external validation, as similar findings in metabolomics studies have been attributed to potential overfitting. In contrast, the LASSO model yielded slightly lower but more conservative performance, supporting the stability of the selected features.

Importantly, despite these considerations, several lines of evidence support the reliability of our findings. First, the consistent identification of overlapping metabolites across three independent algorithms suggests that these features are not driven by a single model but reflect relatively stable biological signals within the current dataset. Second, the majority of the selected metabolites demonstrated moderate-to-high individual discriminatory power within the current cohort, further supporting their relevance. Third, the observed metabolic patterns are consistent with our pathway enrichment results showing significant involvement of amino acid metabolism (e.g., tryptophan, phenylalanine, and methionine pathways), as well as the identification of microbiota-related metabolites such as Trimethylamine N-oxide and indoxyl sulfate, together supporting the biological relevance of these alterations in cardiovascular disease.

Taken together, these results indicate that the identified 14-metabolite signature retains strong discriminative capacity and robustness within internal validation based on cross-validation, while requiring validation in independent external cohorts to confirm its generalizability.

### Gut microbiota–derived metabolites as central drivers of AS

Among the 14 key metabolites, several are derived from gut microbiota and have been widely implicated in cardiovascular disease. Trimethylamine N-oxide (TMAO) emerged as the most prominent feature, showing the highest diagnostic performance and consistent importance across models. TMAO is generated from dietary precursors such as choline and carnitine through microbial metabolism followed by hepatic oxidation, and has been shown to promote foam cell formation, enhance platelet activation, and impair endothelial function (19, 33, 34). Elevated circulating TMAO levels have been consistently associated with increased risk of atherosclerotic events.

Indoxyl sulfate, another microbiota-derived metabolite originating from tryptophan metabolism, is a well-known uremic toxin that induces oxidative stress and vascular inflammation (12). Its identification in the present study further supports the contribution of microbial metabolites to vascular dysfunction.

These findings collectively suggest that microbiota-derived metabolites, rather than microbial composition changes alone, may act as important functional mediators in AS and may serve as functional mediators linking the gut microbiome to vascular disease.

### Amino acid metabolic imbalance and immune–vascular regulation

A second major group of metabolites is related to amino acid metabolism, including tryptophan, phenylalanine, methionine, and their derivatives such as 2-aminobutyric acid and 2-aminoisobutyric acid. These metabolites are closely associated with immune regulation, oxidative stress, and endothelial function (35–38).

Notably, tryptophan did not exhibit a statistically significant difference between groups but demonstrated strong diagnostic performance, suggesting that its role in AS may depend on metabolic flux or downstream pathway activity rather than absolute concentration. This observation is consistent with previous studies showing that the balance between tryptophan and its metabolites is critical for regulating immune responses.

Phenylalanine accumulation has been associated with impaired nitric oxide bioavailability and increased oxidative stress (39, 40), while methionine metabolism influences redox homeostasis (41). Together, these alterations suggest that amino acid metabolic imbalance may contribute to vascular dysfunction and immune dysregulation in AS.

Recent evidence has further highlighted the importance of impaired macrophage efferocytosis in atherosclerotic plaque progression. Defective clearance of apoptotic cells contributes to necrotic core formation, unresolved inflammation, and plaque instability. Notably, RBPJ-mediated epigenetic regulation has been reported to enhance macrophage efferocytosis through modulation of H3K9me3-dependent transcriptional programs, thereby promoting apoptotic cell clearance and suppressing inflammation (42). These findings suggest that metabolic dysregulation and immune dysfunction may converge on impaired efferocytosis pathways, ultimately contributing to chronic vascular inflammation and progression of AS.

### Lipid and bile acid metabolism in AS progression

Notably, recent evidence has suggested that macrophage metabolic reprogramming, particularly enhanced fatty acid synthesis, can promote pathogenic fibroblast expansion and contribute to vascular remodeling in atherosclerotic lesions, highlighting a functional link between lipid metabolic states in macrophages and structural remodeling of the vascular wall (43–45).

Lipid-related and bile acid–related metabolites also played important roles among the identified signatures. Cholesteryl sulfate was uniquely elevated in HC, suggesting a potential protective role. Previous studies have indicated that cholesteryl sulfate contributes to membrane stability and may modulate lipid signaling and inflammatory responses, and its reduction may be associated with vascular dysfunction (46).

In contrast, lithocholic acid 3-sulfate, a secondary bile acid derivative (47, 48), reflects gut microbial activity and host detoxification processes. Bile acids are increasingly recognized as signaling molecules that regulate metabolism and inflammation through nuclear receptors such as FXR and TGR5 (49, 50). Therefore, alterations in bile acid metabolism may have important implications for vascular homeostasis and disease progression.

### Functional rather than compositional alterations in gut microbiota

Despite the significant metabolic differences observed, gut microbiota composition showed only modest changes between AS and HC. Alpha- and beta-diversity analyses did not reveal significant differences, and the dominant taxa were largely similar between groups. This finding is consistent with emerging evidence that functional alterations in microbial metabolism may be more important than compositional shifts in chronic metabolic and cardiovascular diseases.

Indeed, correlation analysis revealed significant associations between key metabolites and specific bacterial genera, including Roseburia, Mediterraneibacter, and unclassified RF39. These results suggest that specific microbial taxa may be involved in shaping host metabolic profiles through the production or modulation of bioactive metabolites, rather than directly reflecting compositional dysbiosis. This supports the concept of a microbiome–metabolite interaction axis in AS within the context of an observational association framework.

### Linking metabolic alterations to cellular senescence

An important finding of this study is the incerased transcriptional expression of senescence-associated markers P16 and P21 in circulating PBMCs from AS patients, suggesting the presence of senescence-related transcriptional alterations in peripheral immune cells. However, these observations are based on mRNA expression levels in PBMCs and do not directly reflect protein expression or cellular senescence status in vascular tissues or plaque-resident cells.

Cellular senescence has been implicated in atherosclerosis progression through its effects on vascular dysfunction and inflammatory remodeling. In this context, our findings may reflect systemic immune cell–associated senescence signals rather than local vascular senescence within the arterial wall.

The observed correlations between P21 and cholesteryl sulfate, as well as between P16 and Escherichia–Shigella, suggest potential associations among metabolic alterations, microbial factors, and senescence-related transcriptional changes in circulating immune cells within the current cohort However, given the cross-sectional design of this study, these relationships should be interpreted as associative signals rather than causal interactions.

Notably, classical inflammatory markers did not show significant differences between groups, indicating that senescence-associated transcriptional alterations in PBMCs may occur independently of overt systemic inflammatory changes in peripheral circulation.

Overall, these results suggest that peripheral blood–derived senescence-associated transcriptional signals may be associated with metabolic and microbiome alterations in AS, while further validation at the protein level and in vascular tissues is required to determine their functional relevance.

### Clinical implications and future perspectives

To contextualize the multi-omics findings, we systematically evaluated key demographic and clinical characteristics of the study cohort. As summarized in Table 1, variables including age, sex, BMI, lipid profile, glycemic status, and major comorbidities were recorded and compared between groups. Although modest differences were observed in age, BMI, and hypertension status, most biochemical parameters were broadly comparable, suggesting an overall relatively balanced baseline for downstream analyses.

Participants with recent antibiotic exposure were excluded during recruitment to minimize its well-established impact on gut microbial composition. However, detailed information regarding dietary patterns and medication stratification (e.g., statins, antihypertensive or antiplatelet therapies) was not fully available, representing a limitation of the current study.

Despite these considerations, metabolomic and microbiome signatures were highly consistent across analytical layers and aligned with established biological pathways implicated in atherosclerosis. Accordingly, while residual confounding from unmeasured clinical variables cannot be completely excluded, the primary findings are unlikely to be solely driven by baseline clinical differences. These results therefore represent robust exploratory associations that warrant validation in larger, well-annotated independent cohorts. Beyond their biological implications, the 14 metabolites identified in this study demonstrate strong potential as diagnostic biomarkers for AS within the current cohort. Their consistent performance across multiple machine learning models highlights their robustness and potential utility in clinical settings. In addition, several of these metabolites, particularly those derived from gut microbiota, are modifiable through dietary interventions, microbiome-targeted therapies, or pharmacological approaches.

However, several limitations should be considered. The relatively small sample size may increase the risk of overfitting despite the use of internal cross-validation, and the cross-sectional design limits causal interpretation. Furthermore, functional validation of the identified metabolite–microbiome interactions is required. This study should therefore be interpreted as an exploratory analysis based on internal cross-validation, and independent external validation will be necessary to confirm the generalizability of the findings.

Future studies should focus on validating these findings in larger cohorts, exploring longitudinal changes in metabolite profiles, and conducting mechanistic experiments to elucidate the causal relationships among microbiota, metabolism, and vascular pathology.

## Conclusion

In summary, this study identifies a robust panel of 14 metabolite signatures that distinguish AS from healthy controls and reveals coordinated alterations in metabolic pathways, gut microbiota function, and cellular senescence. These findings provide new insights into the metabolic basis of AS and suggest that targeting metabolic and microbiome-related pathways may represent a promising strategy for disease diagnosis and intervention.

## Data Availability

The raw data supporting the conclusions of this article will be made available by the authors, without undue reservation.

## Supplementary material

Supplementary Figure S1 Validation of inflammatory markers by qPCR and ELISA. **(A)** Relative mRNA expression levels of P65 and Caspase-1 in AS and HC groups. Bars represent group means, and overlaid dots represent individual samples. Statistical significance was assessed using Student's *t*-test. **(B)** Serum levels of inflammatory cytokines measured by ELISA. Bars represent group means, and overlaid dots represent individual samples. Statistical significance was assessed using Student's *t*-test. IL-18 and IL-6 tended to be higher in HC, whereas MCP-1 was elevated in AS; however, all differences were not statistically significant.
