# Supplementary figures and images for: Integrative multi-omics analysis identifies a robust 14-metabolite signature and reveals microbiome–metabolite–host interactions in atherosclerosis
Source: Front Cardiovasc Med. 2026 Jun 8;13:1849138. doi: 10.3389/fcvm.2026.1849138 (PMC13283812; doi:10.3389/fcvm.2026.1849138)

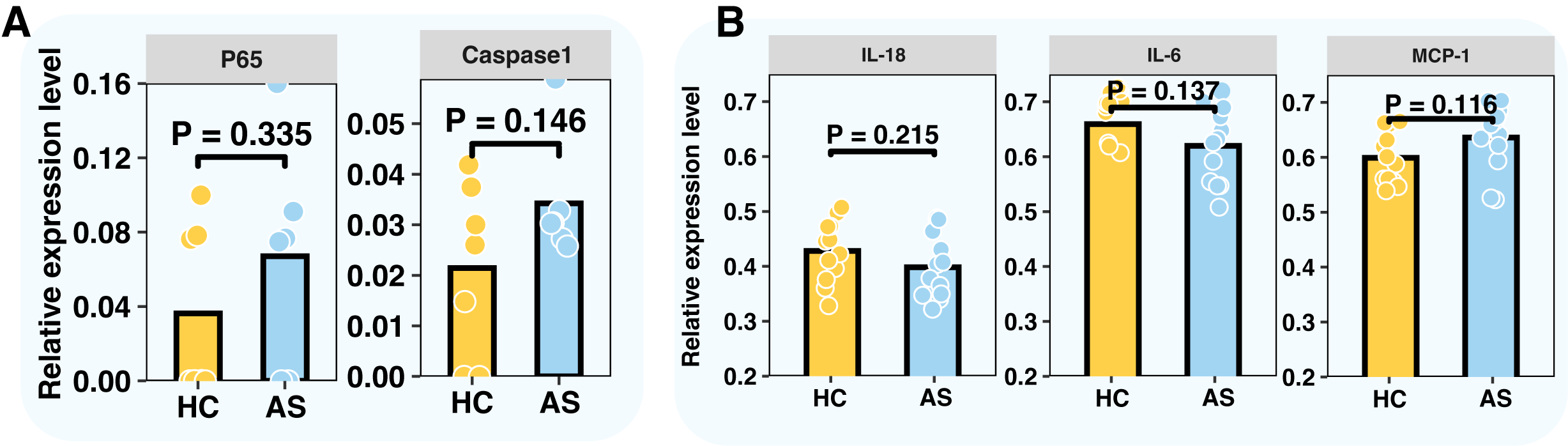

Supplement: Supplementary Figure S1 — Validation of inflammatory markers by qPCR and ELISA. (A) Relative mRNA expression levels of P65 and Caspase-1 in AS and HC groups. Bars represent group means, and overlaid dots represent individual samples. Statistical significance was assessed using Student's t-test. (B) Serum levels of inflammatory cytokines measured by ELISA. Bars represent group means, and overlaid dots represent individual samples. Statistical significance was assessed using Student's t-test. IL-18 and IL-6 tended to be higher in HC, whereas MCP-1 was elevated in AS; however, all differences were not statistically significant. [file Image1.tif]
